# Multisystem Inflammatory Syndrome Presenting as Early Acute Appendicitis

**DOI:** 10.7759/cureus.20200

**Published:** 2021-12-06

**Authors:** Jonathan Anderson, Donna Bhisitkul, Tuan Pham, Kayla Wilson, Andrew R Barbera

**Affiliations:** 1 Department of Emergency Medicine, Lakeland Regional Health, Lakeland, USA; 2 Department of Pediatric Emergency Medicine, Lakeland Regional Health, Lakeland, USA; 3 Department of Pediatric Surgery, Nemours Children's Health System, Orlando, USA; 4 Department of Pharmacy, Lakeland Regional Health, Lakeland, USA

**Keywords:** mis-c, covid-19, acute care surgery, gastrointestinal, pediatric surgery

## Abstract

An 11-year-old male presented to the pediatric emergency department with a one-day history of peri-umbilical pain with nausea, anorexia, and scant vomiting. On examination, he had moderate tenderness in the right upper quadrant with moderate guarding and rebound tenderness. Imaging showed concern for early acute appendicitis. The patient was admitted and underwent laparoscopic appendectomy. Despite the appendectomy, the patient continued to have fevers and abdominal pain. Four days after the initial presentation, the patient decompensated and was diagnosed with multisystem inflammatory syndrome. This case is interesting because the patient never met diagnostic criteria for multisystem inflammatory syndrome in children (MIS-C) prior to his decompensation. If a patient’s symptoms continue or worsen despite seemingly appropriate management, the patient must be reassessed for other causes of pathology. Surgeons must have a high index of suspicion for MIS-C in patients with recent COVID-19 diagnoses, and this case demonstrates that MIS-C can present in phases and not all at once.

## Introduction

Multisystem inflammatory syndrome in children (MIS-C) is a rare complication following coronavirus disease 2019 (COVID-19) illness, in which a subset of patients develop a hyperinflammatory state progressing to severe organ dysfunction. MIS-C is defined by the Centers for Disease Control and Prevention (CDC) as a constellation of six principal elements in a patient less than 21 years old: fever ≥ 38°C for ≥24 hours (or report of subjective fever lasting at least 24 hours), laboratory evidence of inflammation, evidence of clinically severe illness requiring hospitalization, multisystem (≥2) organ involvement, no alternative plausible diagnosis, and recent severe acute respiratory syndrome coronavirus 2 (SARS-CoV-2) infection or confirmed exposure within four weeks prior to symptom onset [1]. Abdominal pain is a common presenting symptom, but patients typically present with some combination of high fever for multiple days, rash, conjunctivitis, mucosal changes, lymphadenopathy, and/or neurologic symptoms. Patients can deteriorate rapidly despite adequate fluid resuscitation with hypotension, multisystem organ failure, and shock requiring vasopressor and intensive care support. Currently recommended treatment options include empiric antibiotics until sepsis can be ruled out, steroids, intravenous immunoglobulin (IVIG), aspirin, and anticoagulation. This report describes the case of MIS-C presenting as early acute appendicitis to the pediatric emergency department (ED).

## Case presentation

An 11-year-old male with a past medical history of asthma and intussusception at age three presented to the pediatric emergency department with a one-day history of peri-umbilical pain with nausea, anorexia, and scant vomiting. The patient had been diagnosed with COVID-19 four weeks prior with symptoms of cough, myalgia, and loss of smell and taste lasting for 11 days. The patient's symptoms abated with supportive care only. At the time of diagnosis, the patient's mother and older brother were also positive. On presentation, the patient was afebrile, normotensive, and mildly tachycardic. Additionally, the family denied any history of fever prior to arrival. Physical exam was significant for moderate tenderness in the right upper quadrant with guarding, and mild rebound tenderness. Laboratory studies were significant for white blood cell (WBC) count: 8.6 k/µL (4.5-13.5), absolute lymphocyte count (ALC): 0.6 k/µL (0.90-3.90), platelets: 320 k/µL (150-450), C-reactive protein (CRP): 2.3 mg/dL (0-0.29), and erythrocyte sedimentation rate (ESR): 29 mm/hr (0-15) (Table 1).

**Table 1:** Laboratory studies. WBC: white blood cells; ALC: absolute lymphocyte count; CRP: C-reactive protein; ESR: erythrocyte sedimentation rate; BNP: B-type natriuretic peptide.

	ED visit #1	ED visit #2	Reference range		ED visit #3	Reference range
WBC	8.6 k/µL	4.6 k/µL	4.5–13.5		6.6 k/µL	4.5–13
ALC	0.6 k/µL	0.4 k/µL	0.9–3.9		0.22 k/µL	0.7–7.1
Platelets	320 k/µL	234 k/µL	150–450		88 k/µL	140–450
CRP	2.3 mg/dl	4.3 mg/dl	0–0.29		13.7 mg/dl	0–0.3
ESR	29 mm/hr	–	0–15		–	–
Bilirubin	0.4 mg/dL	–	0.2–1		2.3 mg/dL	0.2–1
Albumin	4.1 gm/dL	–	3.4–5		2.5 gm/dL	3.4– 5
D-dimer	–	–	–		5,800 ng/mL	0–1,500
Troponin	–	–	–		0.015 ng/mL	0–0.045
BNP	–	–	–		109 pg/mL	0–100

**Figure 1:**
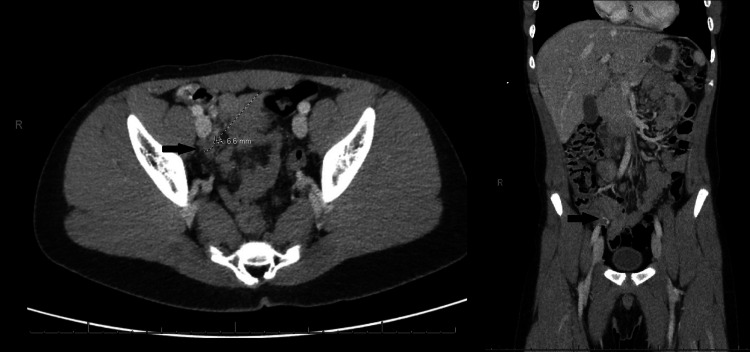
CT showing a 7-mm fluid-filled appendix with a “mildly enhancing” wall and small free fluid.

A right upper quadrant abdominal ultrasound (US) was performed and revealed a positive Murphy’s sign. The appendix was not visualized on the US; however, scattered lymphadenopathy was present, measuring up to 13 mm. A CT scan of the abdomen and pelvis was performed and demonstrated a 7-mm fluid-filled appendix and a small amount of free fluid (Figure 1). The wall of the appendix was described as “mildly enhancing.” The radiologist’s findings were “not convincing for definite acute appendicitis; however, early acute appendicitis cannot be excluded.”

Pediatric surgery was consulted and evaluated the patient. The patient developed his first fever of 39.6°C five hours after presentation. Given worsening pain, fever, and clinical presentation, the patient was taken to the operating room for laparoscopic appendectomy. The appendix was visualized and described as “indurated near the tip with mild erythema.” An appendectomy was performed and no complications were noted. The patient had an uncomplicated overnight stay and was discharged home the following morning.

The patient returned to the pediatric ED 18 hours after discharge for evaluation of fever and continued abdominal pain. On presentation, the patient had a temperature of 38.5°C, heart rate of 105 bpm, respiratory rate of 26 br/min, and blood pressure of 129/76 mmHg. Physical exam was positive for slight abdominal distension with mild diffuse tenderness. The patient received prompt fluid resuscitation with 20 mL/kg of normal saline. Laboratory studies revealed a WBC of 4.6 k/µL (4.5-13.5), ALC of 0.40 k/µL (0.90-3.90), platelet level of 234 k/µL (150-450), and CRP level of 4.3 mg/dl (0-0.29) (Table 1). Pediatric surgery was again consulted and the patient was admitted for observation and close monitoring. Empiric antibiotics were initiated and the patient received ketorolac and morphine for analgesia. A repeat abdominal US revealed a lymph node measuring 1.3 x 1.9 cm with no free fluid or visualized abscess. During this admission, the pathology report was finalized from his prior appendectomy and was finalized as “benign… with focal mucosal acute inflammation, lymphoid follicular hyperplasia, and subserosal vascular congestion.” The patient symptomatically improved and was discharged the following afternoon with a presumptive diagnosis of mesenteric adenitis, post-procedural fever, and abdominal pain.

On the afternoon of discharge, the patient began to clinically deteriorate and his fever increased to 39.4°C at home. The patient presented to an outside pediatric ED and was found to be ill-appearing and lethargic with a diffuse macular rash and bilateral conjunctivitis. The patient had an episode of hypotension (92/51 mmHg) in the ED that responded to fluid resuscitation. Initial laboratory values were as follows: WBC: 6.6 k/µL, ALC: 0.22 k/µL, platelets: 88 k/µL, CRP: 13.7 mg/dL, d-dimer: 5,800 ng/mL, bilirubin: 2.3 mg/dL, albumin: 2.5 gm/dL, troponin: 0.015 ng/mL, and B-type natriuretic peptide (BNP): 109 pg/mL (Table 1). A chest X-ray demonstrated right basilar perihilar opacity with suspected small bilateral pleural effusions.

The patient was admitted for treatment of presumptive MIS-C with cardiology and infectious disease consultations. Treatment was initiated with prophylactic enoxaparin, aspirin, methylprednisolone, and IVIG. An echocardiogram was consistent with borderline ectasia of the right and left main coronary arteries. The patient tested negative on COVID-19 polymerase chain reaction (PCR) and positive for both anti-COVID-19 immunoglobulin M (IgM) and immunoglobulin G (IgG) antibodies. With continued therapy, the patient’s symptoms abated along with decreasing inflammatory markers and rising platelets. The patient was discharged on hospital day four with a discharge plan of aspirin and enoxaparin, along with a four-week prednisone taper.

## Discussion

Gastrointestinal (GI) symptoms predominate in MIS-C and have been reported to occur in nearly 92% of patients [2]. These GI symptoms may mimic many infectious or inflammatory etiologies or, as in our case, require surgical intervention for suspected acute appendicitis. A recent case report describes MIS-C presenting with signs and symptoms of appendicitis; however, this case patient also met MIS-C criteria on presentation [3]. Fever is the key diagnostic criteria in MIS-C as evidenced by a large *Lancet* review of 662 patients, with 100% of patients presenting with fever and a mean duration of fever before the presentation of 4.8 days [4].

**Figure 2:**
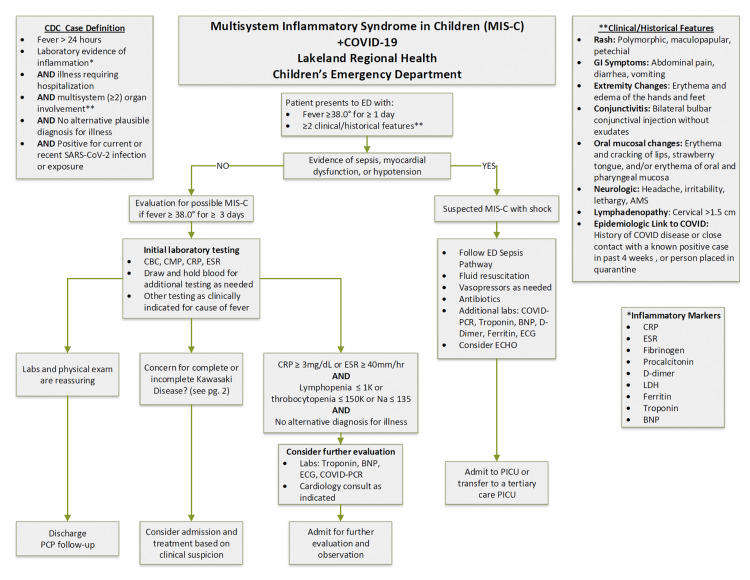
Lakeland Regional Health MIS-C screening algorithm. MIS-C: multisystem inflammatory syndrome in children; SARS-CoV-2: severe acute respiratory syndrome coronavirus 2; PCP: primary care physician; CBC: complete blood count; CMP: comprehensive metabolic panel; CRP: C-reactive protein; ESR: erythrocyte sedimentation rate; BNP: B-type natriuretic peptide; COVID-19: coronavirus disease 2019; PCR: polymerase chain reaction; PICU: pediatric intensive care unit; ECHO: echocardiogram; AMS: altered mental status; LDH: lactate dehydrogenase.

Our clinical pathway (Figure 2) utilizes fever plus two clinical/historical features, including the history of COVID-19 illness or close contact with a known positive case in the previous four weeks as criteria to consider MIS-C. Our patient presented without fever and subsequently decompensated four days after the initial presentation to the ED.

## Conclusions

Our case demonstrates that signs and symptoms of MIS-C can present in many phases and not all at once. If the patient’s clinical condition deteriorates despite appropriate management, a diagnosis of MIS-C must be revisited. MIS-C should also be considered earlier if the patient appears septic or ill-appearing on initial presentation, which our patient did not. With appropriate therapy and prompt recognition, MIS-C is treatable and most children will have a good outcome. Fortunately, our patient returned for emergency medical care, received appropriate and prompt therapy, and is now doing well.
